# Prognostic value of Dickkopf-1 and ß-catenin expression in advanced gastric cancer

**DOI:** 10.1186/s12885-018-4420-8

**Published:** 2018-05-02

**Authors:** Soon Auck Hong, Su Hyun Yoo, Han Hong Lee, Der Sheng Sun, Hye Sung Won, Okran Kim, Yoon Ho Ko

**Affiliations:** 1 Department of Pathology, Soonchunhyang University Cheonan Hosptial, Cheonan, Republic of Korea; 2 Medical Clinic Laboratory Department of U2Bio Co. Ltd., Seoul, Republic of Korea; 3 Department of General Surgery, College of Medicine, The Catholic University of Korea, Seoul, Republic of Korea; 4 Division of Oncology, Department of Internal Medicine, College of Medicine, The Catholic University of Korea, 222, Banpodaero, Seochogu, Seoul, 06591 Republic of Korea; 5 Cancer Research Institute, College of Medicine, The Catholic University of Korea, Seoul, Republic of Korea

**Keywords:** Dickkopf-1, Gastric cancer, Prognosis, Cut value

## Abstract

**Background:**

Dickkopf-1 (DKK1) is a Wnt/ß-catenin pathway antagonist related to gastric cancer (GC) carcinogenesis. However, the prognostic role of combined DKK1 and ß-catenin expression in advanced GC (AGC) is not clear.

**Methods:**

In total, 158 patients with AGC who underwent gastric resection were enrolled in this study. DKK1 and ß-catenin expression was evaluated in whole tumor sections by immunohistochemistry.

**Results:**

DKK1 expression was high in 73 (46.2%) patients, while ß-catenin expression was positive in 51 (32.3%) patients. The expression of DKK1 was positively correlated with that of ß-catenin (*P* < 0.001). The combined expression of DKK1 and ß-catenin was significantly associated with high N stage (N2 and N3) (*P* = 0.042). In addition, patients with high DKK expression demonstrated poorer overall (OS) (*P* < 0.001) and disease-free survival (DFS) (*P* = 0.001). However, there were no differences between high DKK1 expression with ß-catenin positivity and high DKK1 expression with ß-catenin negativity (OS, *P* = 0.379: DFS, *P* = 0.255). Multivariate analysis revealed that high DKK1 alone or high DKK1 with ß-catenin positivity were independent prognostic factors for both OS (high DKK1: hazard ratio [HR], 2.130; 95% confidence interval [CI]; 1.370–3.312, *P* = 0.001; high DKK1 with ß-catenin positivity: HR, 2.140; 95% CI, 1.343–3.409: *P* = 0.001) and DFS (high DKK1: HR, 2.092; 95% CI, 1.180–3.708; *P* = 0.012; high DKK1 with ß-catenin positivity: HR, 2.357; 95% CI, 1.291–4.306; *P* = 0.005).

**Conclusion:**

Our results indicate that high DKK1 expression regardless of ß-catenin positivity is a crucial prognostic factor for predicting tumor recurrence and survival in patients with resected AGC.

**Electronic supplementary material:**

The online version of this article (10.1186/s12885-018-4420-8) contains supplementary material, which is available to authorized users.

## Background

Gastric cancer is the fourth most commonly diagnosed cancer and the second most common cause of cancer-related death worldwide [1]. Recently, the development of surgical techniques and target therapies has led to a significant improvement in survival. Currently, early gastric carcinoma (EGC) has a 5-year survival rate of over 90% [2]. However, for cases of advanced GC (AGC), the survival rate is still around 40% [3]. Thus, a new biomarker for AGC is urgently needed.

The Wnt signaling cascade governs cell proliferation, cell polarity, and cell fate during embryonic development and homeostasis in human tissues [4]. Solid tumors frequently exhibit dysregulated Wnt signaling pathways, and this dysregulation is linked to enhanced malignant potential [5]. In GC, activation of the Wnt/ß-catenin pathway is found in approximately 30% to 50% of tumors [6, 7]. The first step in activation is the binding of the Wnt ligand to the seven-pass transmembrane Frizzled receptor (FZ), low-density lipoprotein receptor-related protein 6 (LRP 6), and LRP 5. The Wnt-FZ-LRP5/6 complex disrupts Axin-mediated ß-catenin phosphorylation, resulting in ß-catenin stabilization. Accumulation of ß-catenin in the cytoplasm results in its transport to the nucleus, where ß-catenin forms complexes with T-cell factor (TCF)/lymphoid enhancer factor and activates Wnt-targeted gene expression (cyclin D1, *c-MYC*, axin-2) [5, 8]. Wnt signaling can be repressed by six Wnt antagonist families, including Dickkopf (DKK) proteins, secreted Frizzled-related proteins, WNT-inhibitory factor 1, Wise/SOST, Cerberus, and insulin-like growth-factor binding protein 4 [9]. DKK1, the most well-known Wnt antagonist, is a 35 kDa protein that contains a secreted signal peptide sequence [10]. Antagonism of the Wnt pathway via antagonists such as DKK1 is accomplished through binding to LRP 5/6 [11, 12]. Despite being a negative regulator of Wnt signaling, the prognostic role of DKK1 is not well understood. Results from previous studies on DKK1 in diverse tumors have demonstrated conflicting results; some have reported that DKK1 acts as a tumor suppressor, while others have shown that it acts as an oncogene [13]. Lee et al. and Gao et al. reported that overexpression of DKK1 protein and mRNA in tissue and increased levels of DKK1 in serum were significantly associated with unfavorable prognosis in patients with GC [14, 15]. In contrast, other recent studies showed low DKK1 expression in GC samples, and that restoration of DKK1 in tumor cells inhibited tumor cell growth and invasion [16, 17]. However, the prognostic value of DKK1 in GC is not clearly determined. Furthermore, although DKK1 interacts with ß-catenin, the association between DKK1 and abnormal ß-catenin expression has not been well studied. Therefore, we aimed to confirm the role of DKK1 as a prognostic factor in AGC and to determine the association between DKK1 and ß-catenin in a sizable AGC cohort using immunohistochemistry.

## Methods

### Study population

The clinical and pathological data of 158 patients with AGC who had undergone primary radical resection between 2001 and 2005 at Uijeongbu St. Mary’s Hospital of the Catholic University of Korea were reviewed. The inclusion criteria were (i) pathologically confirmed adenocarcinoma, (ii) radical resection without preoperative radiation or chemotherapy, (iii) removal of at least 15 or more lymph nodes, and (iv) available paraffin-embedded tumor specimens. Postoperative pathological staging was based on the American Joint Committee on Cancer (AJCC) staging criteria, 7th edition. This study was approved by the Institutional Research Ethics Board of Uijeongbu St. Mary’s Hospital of the Catholic University of Korea and adhered to the Declaration of Helsinki. Patient anonymity was preserved.

### Immunohistochemistry

Immunohistochemistry was performed on formalin-fixed paraffin-embedded tissue sections. Whole tissue sections of representative tumor samples were deparaffinized using xylene and graded alcohol and then rehydrated with distilled water. Blocking of endogenous peroxidase activity was achieved by quenching with 3% hydrogen peroxide in methanol for 10 min. Antigen retrieval was then performed by heating the slides for 15 min in 0.01 M citrate buffer (pH 6.0). The sections were incubated with human specific antibodies against DKK1 (1:200, Abcam, Cambridge, UK) and ß-catenin (1:100, Cell Signaling, Danvers, MA, USA) at room temperature for 30 min, washed in phosphate-buffered saline, and then incubated with a peroxidase-labeled polymer conjugated to the secondary antibody for 30 min. The immunoreaction was visualized using the Polink-2 plus polymer HRP detection system (Golden Bridge International, Mukilteo, WA, USA). In the negative controls, the primary antibodies were substituted with normal rabbit or mouse IgG at the same concentration as the primary antibody. Immunohistochemical staining was independently examined by two board-certified pathologists (S.A.H. and S.H.Y.) who were blinded to the clinical data. The intensity of DKK1 staining was scored using the following scale: 0, no staining; 1, weakly positive; 2, moderately positive; 3, strongly positive. The proportion of DKK1 staining was calculated as the percentage of positive tumor cells. H scores, ranging from 0 to 300, were determined by multiplying the intensity score by the proportion. The cut-off H score for high DKK1 expression was set at 60 using maximally selected rank statistics (maxstat) calculated in R statistical programming language version 3.2.3 (https://www.r-project.org/). ß-catenin expression was considered positive if more than 10% of tumor cells showed nuclear and cytoplasmic staining [18].

### Statistical methods

Chi-square or Fisher’s exact tests were conducted to determine the association among DKK1 expression, ß-catenin expression, and clinicopathologic parameters. A *P*-value of < 0.05 was considered statistically significant. Overall survival (OS) was calculated from the date of diagnosis to the date of death due to any cause or the last follow-up visit. Disease-free survival (DFS) was calculated from the date of diagnosis to the date of the first distant or local disease recurrence or last follow-up. The Kaplan-Meier method was used to analyze ‘time-to-event’ data, and statistical differences in the cumulative survival curves were evaluated using the log-rank test. Cox proportional hazards regression models were used to investigate the significance of the prognostic factors. All variables with a *P*-value < 0.05 in the univariate analysis were included in the multivariate analysis. Survival rates and hazard ratios are shown with their respective 95% confidence intervals (CIs). Statistical analyses were performed using R statistical programming language version 3.2.3 (https://www.r-project.org) and the SPSS software package (version 18.0; SPSS, Chicago, IL, USA).

## Results

### Patients

Patient age at diagnosis ranged from 29 to 89 (median: 62) years. The patient cohort consisted of 113 males and 45 females. Based on the AJCC staging criteria, 18 patients (11.4%) had stage I disease, 50 (31.6%) had stage II disease, 88 (55.7%) had stage III disease, and 2 (1.3%) had stage IV disease. Ninety-seven (61.4%) patients received 5-fluorouracil and cisplatin combination therapy postoperatively. The follow-up period ranged from 0 to 152.0 months, with a median of 68.0 months, after curative surgical resection. Of the 158 total patients, 91 (57.6%) died, and 67 (42.4%) were alive at the last follow-up. Disease recurrence was observed in 52 cases (32.9%).

### DKK1 and ß-catenin expression in advanced GC

**Fig. 1:**
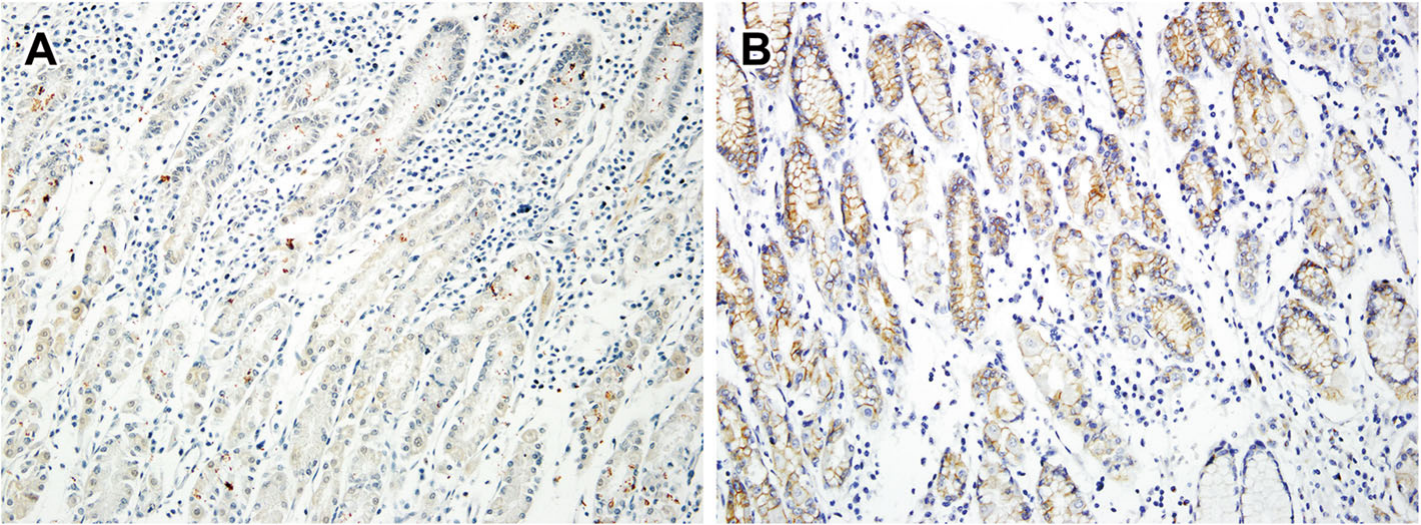
Immunohistochemical staining of DKK1 and β-catenin in normal gastric mucosa. Scant expression of DKK1 (**a**) and membranous staining (**b**) of β-catenin were observed

**Fig. 2:**
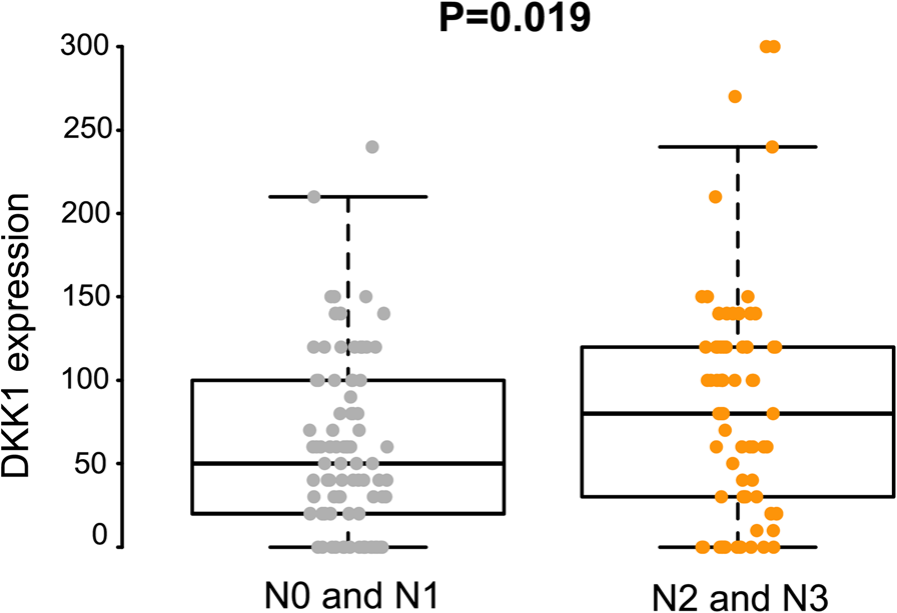
Expression of DKK1 protein in advanced gastric cancer with N1 and N2 is higher than that in N2 and N3

**Fig. 3:**
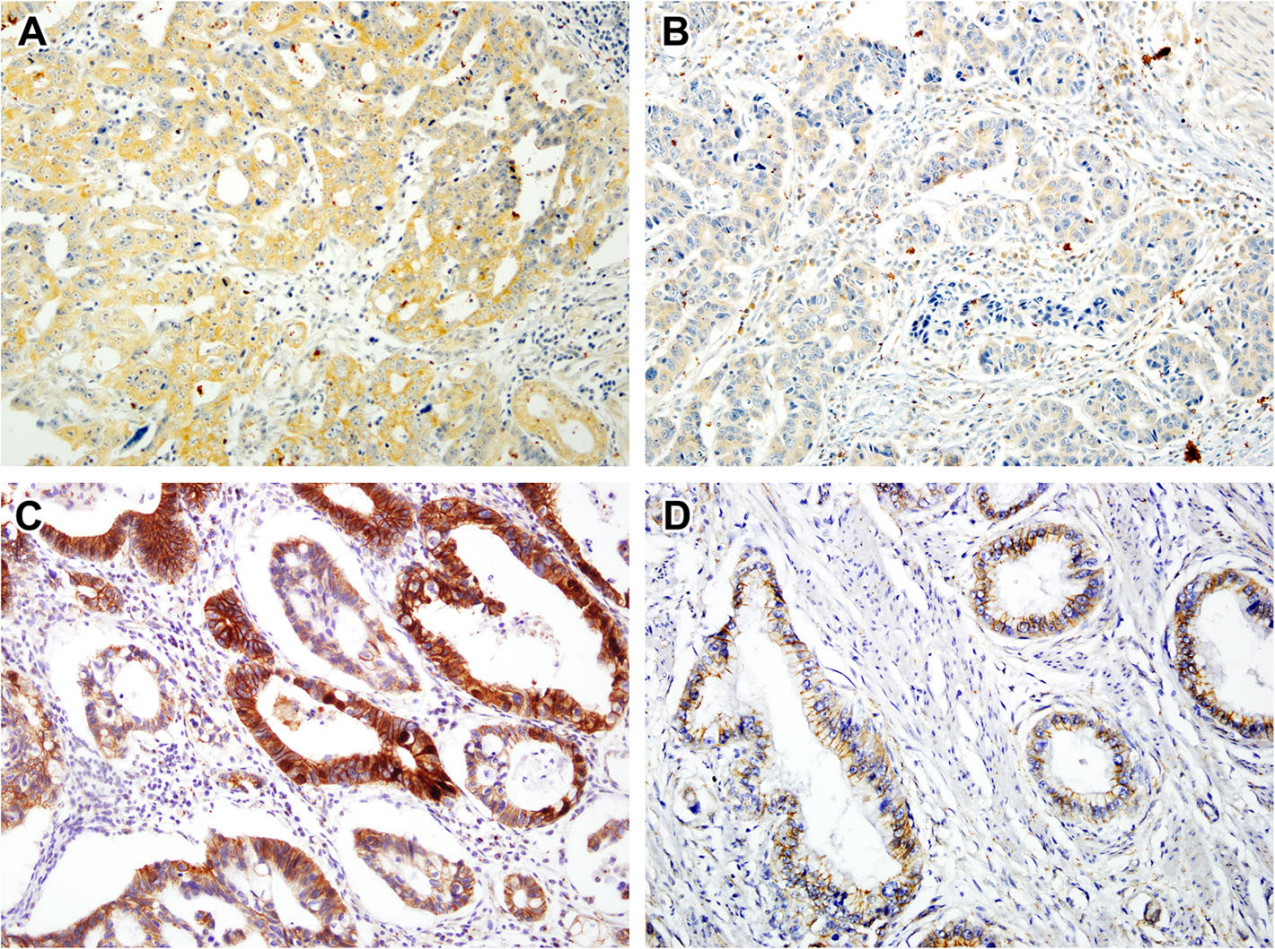
Immunohistochemical staining of DKK1 and β-catenin staining in GC. Representative images of GC with high expression of DKK1 (**a**), low expression of DKK1 (**b**), positive β-catenin staining (**c**), and negative β-catenin staining (**d**)

In non-neoplastic gastric epithelium, only scant or weak cytoplasmic staining of DKK1 was detected (Fig. 1a), while ß-catenin expression was identified as membranous (Fig. 1b). Increased DKK1 expression H score was found in higher nodal-stage tumors (N0/1, 61.72 ± 51.80 [mean ± standard deviation] vs. N2/3, 84.79 ± 70.81; *P* = 0.019) (Fig. 2). Based on the determined cut-off H score for DKK1 (> 60), 73 (46.2%) and 85 (53.8%) patients were classified into high and low expression groups, respectively (Fig. 3a and b). High expression of DKK1 was statistically associated only with advanced nodal stage (N0/1 vs. N2/3, *P* = 0.021) (Table 1). ß-catenin positivity was observed in 51 cases (32.3%) and negativity in 107 cases (67.7%) (Fig. 3c and d). However, ß-catenin positivity was not related to any clinicopathologic factors (Additional file 1: Table S1). A positive correlation between DKK1 and ß-catenin expression was identified (*P* < 0.001) (Table 2). Stratification of patients with both DKK1 and ß-catenin expression was performed as follows: high DKK1 expression with ß-catenin positivity (*n* = 35, 22.2%), high DKK1 expression with ß-catenin negativity (*n* = 38, 24.1%), low DKK1 expression with ß-catenin positivity (*n* = 16, 10.1%), and low DKK1 expression with ß-catenin negativity (*n* = 69, 43.7%). High DKK1 expression combined with ß-catenin positivity was related to high nodal stage (N0/1 vs. N2/3, *P* = 0.042) (Table 3).

**Table 1:** Correlation between clinicopathologic findings and DKK1 expression

Clinicopathologic features	No. of patients (%)	DKK-1 expression		*P*-value
		Low (%) 85 (53.8)	High (%) 73 (46.2)	
Age, years				
> 62	79 (50.0)	36 (42.4)	43 (58.9)	0.038
≤ 62	79 (50.0)	49 (57.6)	30 (41.1)	
Sex				
Male	113(71.5)	60 (70.6)	53 (72.6)	0.780
Female	45 (28.5)	25 (29.4)	20 (27.4)	
T stage				
T2/3	42 (26.6)	24 (28.2)	18 (24.7)	0.612
T4	116 (73.4)	61 (71.8)	55 (75.3)	
N stage				
N0 to N1	87 (55.1)	54 (63.5)	33 (45.2)	0.021
N2 to N3	66 (44.9)	31 (36.5)	40 (54.8)	
Lauren’s classification				
Diffuse type	105(66.5)	60 (70.6)	45 (61.6)	0.235
Non-diffuse type	53(33.5)	25 (29.4)	28 (38.4)	
Tumor grade				
Well/moderate	46 (29.2)	20 (23.5)	26 (35.6)	0.095
Poor	112 (71.8)	65 (76.5)	47 (64.4)	
Lymphatic invasion				
Yes	127 (80.4)	65 (23.5)	62 (84.9)	0.182
No	31 (19.6)	20 (76.5)	11 (15.1)	
Vascular invasion				
Yes	12 (7.6)	5 (5.9)	7 (9.6)	0.381
No	146 (92.4)	80 (94.1)	66 (90.4)	
TNM stage				
I/II	68 (43.0)	41 (51.8)	27 (37.0)	0.155
III/IV	90 (57.0)	44 (48.2)	46 (63.0)

**Table 2:** Correlation between DKK1 and ß-catenin expression

Coexpression		DKK1 expression		
		Low	High	*P* value
ß-catenin	Negative	69 (81.2)	38 (52.1)	< 0.001
	Positive	16 (18.8)	35 (47.9)

**Table 3:** Correlations between clinicopathologic findings and high DKK1 expression with ß-catenin positivity

Clinicopathologic features	High DKK1 and positive ß-catenin		*P*-value
	Yes (%) 35 (22.2)	No (%) 123 (77.8)	
Age, years			
> 62	21 (60.0)	58 (47.2)	0.180
≤ 62	14 (40.0)	65 (52.8)	
Sex			
Male	27 (77.1)	86 (69.9)	0.403
Female	8 (22.9)	37 (30.1)	
T stage			
T2 /3	11 (31.4)	31 (25.2)	0.462
T4	24 (68.6)	92 (74.8)	
N stage			
N0/1	14 (40.0)	73 (59.3)	0.042
N2/3	21 (60.0)	50 (40.7)	
Lauren’s classification			
Diffuse type	20 (57.1)	85 (69.1)	0.186
Non-diffuse type	15 (42.9)	38 (30.9)	
Tumor grade			
Well/moderate	12 (34.3)	34 (27.6)	0.445
Poor	23 (65.7)	89 (72.4)	
Lymphatic invasion			
Yes	28 (80.0)	99 (80.5)	0.949
No	7 (20.0)	24 (19.5)	
Vascular invasion			
Yes	3 (8.6)	9 (7.3)	0.805
No	32 (91.4)	114 (92.7)	
TNM stage			
I/II	14 (40.0)	54 (43.9)	0.681
III/IV	21 (60.0)	69 (56.1)	

### Prognostic value of DKK1 and ß-catenin expression in advanced GC

**Fig. 4:**
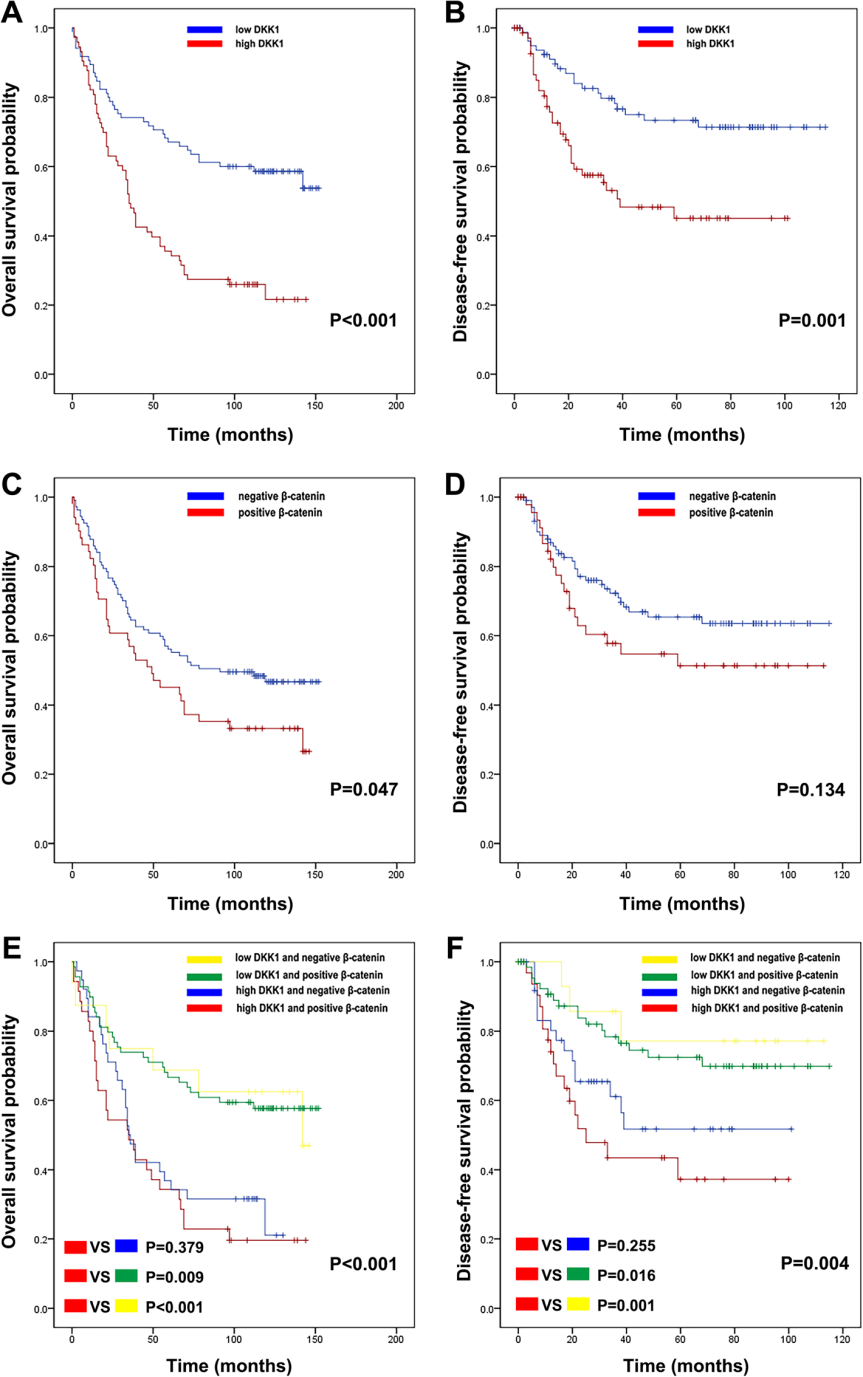
Kaplan-Meier survival curves were plotted according to DKK1 and β-catenin protein expression. High DKK1 expression was significantly related to poor OS (**a**) and DFS (**b**) in GC patients. Patients with positive β-catenin staining showed shorter OS (**c**), while DFS (**d**) was not different significantly between patients with positive and those with negative β-catenin staining. The combination of DKK1 and β-catenin expression was evaluated for its impact on OS (**e**) and DFS (**f**)

We assessed the correlation among DKK1 expression, ß-catenin expression, and prognosis using Kaplan-Meier curves with a log-rank test. High DKK1 expression was significantly associated with shorter OS (*P* < 0.001) and DFS (*P* = 0.001) (Fig. 4a and b). Patients positive for ß-catenin expression showed shorter OS (*P* = 0.047), but there were no differences in DFS between patients positive and those negative for ß-catenin expression (*P* = 0.134) (Fig. 4c and d). Survival analysis of the four groups was performed based on combined DKK1 and ß-catenin expression statuses (Fig. 4e and f). Patients with high DKK1 and ß-catenin positivity demonstrated poorer survival compared with patients with low DKK1 and ß-catenin positivity (OS, *P* = 0.009; DFS, *P* = 0.016) and patients with low DKK1 and ß-catenin negativity (OS, *P* < 0.001; DFS, *P* = 0.001). However, among the patients with high DKK1 expression, no differences were found between those positive and those negative for ß-catenin (OS, *P* = 0.379; DFS, *P* = 0.255). The factors affecting OS and DFS were further analyzed using the Cox proportional hazards regression method. The univariate Cox proportional hazard ratios for OS and DFS were calculated. High DKK1 expression with or without ß-catenin positivity were significantly related with poorer DFS (HR, 2.497; 95% CI, 1.421-4.386; *P* = 0.001) and OS (hazard ratio [HR], 2.509; 95% CI, 1.637-3.846; *P* < 0.001) (Table 4). High DKK1 and ß-catenin positivity was significantly related to unfavorable DFS (HR, 2.512; 95% CI, 1.430–4.413; *P* = 0.001) and OS (hazard ratio [HR], 2.522; 95% CI, 1.645–3.867; *P* < 0.001) (Table 5) and. Results from the multivariate Cox proportional hazards model showed that high DKK1 expression is an independent prognostic indicator for both DFS (HR, 2.092: 95% CI, 1.180–3.708: *P* = 0.012) and OS (HR, 2.130: 95% CI, 1.370–3.312: *P* = 0.001) (Table 4) and. According to combined DKK1 and ß-catenin expression in the multivariate Cox proportional hazard models, high DKK1 and ß-catenin positivity is an independent prognostic value for DFS (HR, 2.357; 95% CI, 1.291-4.306; *P* = 0.005) and OS (HR, 2140; 95% CI, 1.343-3.409; *P* = 0.001) (Table 5).

**Table 4:** Univariate and multivariate analyses of disease-free survival and overall survival using the Cox proportional hazards model for all patients

Characteristics	Univariate analysis of DFS			Multivariate analysis of DFS		
	Hazard ratio	95% CI	*p*-value	Hazard ratio	95% CI	*p*-value
Age (> 62 vs. ≤62)	1.251	0.725 - 2.158	0.420			
Sex (female vs male)	1.005	0.544 - 1.856	0.987			
Advanced T stage (pT4 vs. pT2/3)	7.564	2.356 – 24.286	0.001	6.569	2.014 – 21.422	< 0.001
Advanced N stage (pN2/3 vs. pN0/1)	3.544	1.959 – 6.409	< 0.001	2.500	1.359 – 4.601	0.003
Lauren’s classification (diffuse vs. non-diffuse)	1.255	0.680 - 2.317	0.467			
Venous invasion (yes vs. no)	1.940	0.771 - 4.883	0.159			
Tumor grade (poor vs well to moderate)	1.172	0.626 – 2.197	0.619			
Adjuvant chemotherapy (yes vs. no)	1.196	0.687– 2.083	0.526			
High DKK1 (yes vs. no)	2.497	1.421 – 4.386	0.001	2.092	1.180 – 3.708	0.012
	Univariate analysis of OS			Multivariate analysis OS		
	1.934	1.269 - 2.946	0.002	1.820	1.189-2.785	0.006
	0.656	0.403 - 1.069	0.091			
	3.356	1.825 - 6.170	< 0.001	3.137	1.674 – 5.879	< 0.001
	2.174	1.431 - 3.302	< 0.001	1.661	1.074 – 2.568	0.023
	1.073	0.690 - 1.668	0.755			
	1.763	0.852 - 3.649	0.126			
	1.058	0.671 - 1.668	0.808			
	0.898	0.596 - 1.355	0.610			
	2.509	1.637 – 3.846	< 0.001	2.130	1.370 – 3.312	0.001

**Table 5:** Univariate and multivariate analyses of disease-free survival and overall survival using the Cox proportional hazards model for all patients

Characteristics	Univariate analysis of DFS			Multivariate analysis of DFS		
	Hazard ratio	95% CI	*p*-value	Hazard ratio	95% CI	*p*-value
Age (> 62 vs. ≤62)	1.251	0.725 - 2.158	0.420			
Sex (female vs male)	1.005	0.544 - 1.856	0.987			
Advanced T stage (pT4 vs. pT2/3)	7.564	2.356 – 24.286	0.001	6.569	2.014 – 21.422	< 0.001
Advanced N stage (pN2/3 vs. pN0/1)	3.544	1.959 – 6.409	< 0.001	2.500	1.359 – 4.601	0.003
Lauren’s classification (diffuse vs. non-diffuse)	1.255	0.680 - 2.317	0.467			
Venous invasion (yes vs. no)	1.940	0.771 - 4.883	0.159			
Tumor grade (poor vs well to moderate)	1.172	0.626 – 2.197	0.619			
Adjuvant chemotherapy (yes vs. no)	1.196	0.687– 2.083	0.526			
High DKK1 and positive ß-catenin (yes vs. no)	2.512	1.430 – 4.413	0.001	2.357	1.291 - 4.306	0.005
	Univariate analysis of OS			Multivariate analysis of OS		
	1.934	1.269 - 2.946	0.002	1.820	1.189-2.785	0.006
	0.656	0.403 - 1.069	0.091			
	3.356	1.825 - 6.170	< 0.001	3.137	1.674 – 5.879	< 0.001
	2.174	1.431 - 3.302	< 0.001	1.661	1.074 – 2.568	0.023
	1.073	0.690 - 1.668	0.755			
	1.763	0.852 - 3.649	0.126			
	1.058	0.671 - 1.668	0.808			
	0.898	0.596 - 1.355	0.610			
	2.522	1.645 – 3.867	< 0.001	2.140	1.343 – 3.409	0.001

## Discussion

In this study, high DKK1 expression was significantly associated with high N stage, and patients with high DKK1 expression demonstrated shorter OS and DFS. High DKK1 expression was also correlated with β-catenin positivity. On multivariate analysis, high DKK1 expression and β-catenin positivity were found to be independent adverse prognostic factors for both OS and DFS. Interestingly, patients with high DKK1 expression and concomitant β-catenin negativity also had shorter OS and DFS. Thus, the prognostic value of DKK1 in GC patients may be independent of β-catenin expression.

Various tumors have shown conflicting results regarding the role of DKK1 as a tumor suppressor or oncogene. DKK1 is a secreted protein that plays a crucial role as a negative regulator of the Wnt signaling pathway, and downregulation of DKK1 in colon cancer, breast cancer, hepatocellular carcinoma, and renal cell carcinoma suggests that DKK1 is an antagonist of the Wnt signaling pathway. However, overexpression of DKK1 has been observed in various malignant tumors and is also correlated with adverse prognosis in patients with multiple myeloma, hepatoblastoma, Wilm’s tumor, lung cancer, and breast cancer [19–22].

Although DKK1 has been investigated extensively in various tumors, including GC, no previous studies have evaluated the association between DKK1 and β-catenin expression in AGC. Previous studies have shown that overexpression of DKK1 is related to adverse prognosis in GC [14, 15]. This is consistent with the results we obtained in the high DKK1 expression group, which showed that regardless of β-catenin expression, high DKK1 expression is related to unfavorable prognosis. In previous studies, high DKK1 expression was found to be associated with intestinal GC, advanced T stage, vascular and lymphatic invasion, and distant metastasis. However, we only found a significant association between N stage and high DKK1 expression. This discrepancy might stem from different cut-off values and patient cohorts among studies. Our cut-off value was meticulously determined and optimized for patient survival using maximally selected rank statistics. Therefore, we suggest that our cut-off value better reflects the prognostic role of DKK1 in GC, independently of other adverse clinicopathologic parameters. Previous clinical studies of DKK1 expression in GC included both early GC and AGC, with a number of distant metastasis cases [14, 15]. Survival of early gastric carcinoma patients is over 90%, but survival of patients with both GC and distant metastasis is less than 10% [23]. Therefore, clarification of the prognostic impact of DKK1 could be confounded by the heterogenous patient cohorts used in previous studies.

Wan et al. reported that adenovirus-mediated DKK1 overexpression in CD44^+^ GC cells inhibits tumorigenicity through attenuating Wnt signaling [16]. The results indicate that DKK1 plays a tumor suppressor role in GC stem cells. However, the authors only evaluated the CD44^+^ GC cell line. Although cancer stem cells play a crucial role in GC, they are not entirely representative of GC. Given that the tumor cell, tumor environment, and cell–cell interactions all contribute to GC, studies using limited gastric cancer cell line was further validated.

Activation of Wnt/β-catenin is found in 30% to 50% of GC tissue samples and cell lines [6, 24]. Furthermore, recent studies that used high-throughput sequencing methods showed that *CTNNB1*, which encodes β-catenin, is a driver gene for GC [25–27]. However, *CTNNB1* gene mutations were only detected in 4% to 9% of sporadic GC tumors [28, 29]. These findings suggest that aberrant activation of Wnt signaling pathway is modulated mainly by Wnt ligands and negative regulators, not through mutation of the *CTTNB1* gene. In this study, high DKK1 expression was significantly related to positive β-catenin expression in AGC samples. Given that DKK1 inhibits β-catenin, this finding may seem contradictory. However, several tumor studies revealed that high DKK1 expression is correlated with activation of the Wnt/β-catenin pathway in both hepatocellular carcinoma and hilar cholangiocarcinoma [20, 30]. These findings could be explained by a disruption in the negative feedback loop between DKK1 and the Wnt/β-catenin pathway, which could result from a high level of secreted DKK1 [31]. In addition, DKK1 is also a downstream target gene of β-catenin/TCF, which is a direct target of activated β-catenin [32].

The OS and DFS of patients with high DKK1 and negative β-catenin expression were not different from those with high DKK1 and positive β-catenin expression. These results suggest that the effect of high DKK1 expression in AGC could be an independent of β-catenin status. Conversely, high DKK1 expression, which does not affect canonical Wnt/β-catenin signaling, is still a prognostic factor for patients with AGC.

Our results suggest that high DKK1 expression affects prognosis regardless of β-catenin activation. Several previous studies showed that DKK1 promotes malignancy via non-canonical Wnt pathway mechanisms. In hepatocellular carcinoma, high DKK1 mRNA and protein expression was correlated with poor OS and DFS. In addition, a positive relationship among DKK1 expression, JNK phosphorylation, and RhoA levels was identified [33]. These results indicate that the malignant potential can be increased by the interaction between DKK1 and the non-canonical Wnt pathway, which consists of the Wnt/Ca^2+^ and Wnt/PCP pathways and does not involve activation of β-catenin [34]. Moreover, Kimura et al. reported that cytoskeleton-associated protein 4, a receptor for DKK1, mediates DKK1 signaling to promote cancer cell proliferation via the PI3K/AKT pathway and was associated with an unfavorable prognosis in pancreatic and lung cancer patients [35]. Together, these results suggest that high DKK1 expression acts through β-catenin-independent mechanisms to increase the malignant potential and decrease survival in patients with AGC.

Unfortunately, molecular targeting therapies for AGC are limited to trastuzumab and ramucirumab [36, 37]. Due to the shortage of promising target agents for GC, new targets molecules with potential agents are urgently needed. The efficacy of an anti-DKK1 antibody has been investigated in multiple myeloma and prostate cancers that were associated with bone resorption [38, 39]. However, further preclinical studies to determine the effectiveness of anti-DKK1 antibody in GC are required.

## Conclusions

We found that high DKK1 expression was correlated with a positive β-catenin status. In addition, patients with high DKK1 expression who were positive for β-catenin had a poor prognosis. However, patients with high DKK1 expression who were negative for β-catenin also demonstrated a poor prognosis. In the multivariate analysis, high DKK1 expression only or high DKK1 expression with β-catenin positivity were an independent prognostic factor for OS and DFS in patients with AGC. Together, these results suggest that DKK1 may act as a biomarker and therapeutic target in AGC.

## Additional file

Additional file 1: **Table S1.** Correlation between clinicopathogic findings and ß-cateinin expression. (DOCX 21 kb)

## Abbreviations

AGC: Advanced gastric cancer
CI: Confidence interval
DFS: Disease-free survival
DKK1: Dickkopf-1
EGC: Early gastric cancer
FZ: Frizzled receptor
GC: Gastric cancer
HR: Hazard ratio
LRP: Lipoprotein receptor-related protein
OS: Overall survival
TCF: T-cell factor
