# Supplementary material for: Prognostic value of Dickkopf-1 and ß-catenin expression in advanced gastric cancer
Source: BMC Cancer. 2018 May 2;18:506. doi: 10.1186/s12885-018-4420-8 (PMC5930854; doi:10.1186/s12885-018-4420-8)
Supplement: Supplementary file 1 — Table S1. Correlation between clinicopathogic findings and ß-cateinin expression. (DOCX 21 kb) [file 12885_2018_4420_MOESM1_ESM.docx]

**Additional file 1: Table S1. Correlation between clinicopathogic findings and ß-cateinin expression.**

| Clinicopathologic features | ß-cateinin expression | | *P*-value |
| --- | --- | --- | --- |
|  | Positive (%)  51 (32.3) | Negative (%)  107 (67.7) |  |
| Age |  |  |  |
| >62 | 28 (54.9) | 51 (47.7) | 0.496 |
| ≤62 | 23 (45.1) | 56 (52.3) |  |
| Sex |  |  |  |
| Male | 36 (70.6) | 77 (72.0) | 1.000 |
| Female | 15 (29.4) | 30 (28.0) |  |
| T stage |  |  |  |
| T2 to T3 | 13 (25.5) | 29 (27.1) | 0.850 |
| T4 | 38 (74.5) | 78 (72.9) |  |
| N stage |  |  |  |
| N0 to N1 | 24 (47.1) | 63 (58.9) | 0.175 |
| N2 to N3 | 27 (52.9) | 44 (41.1) |  |
| Lauren's |  |  |  |
| Diffuse type | 30 (58.8) | 75 (70.1) | 0.207 |
| Non-diffuse type | 21 (41.2) | 32 (29.9) |  |
| Tumor grade |  |  |  |
| Well to moderately | 16 (31.4) | 30 (28.0) | 0.710 |
| Poorly | 35 (68.6) | 77 (72.0) |  |
| Lymphatic invasion | |  |  |
| Yes | 41 (80.4) | 86 (80.4) | 1.000 |
| No | 10 (19.6) | 21 (19.6) |  |
| Vascular invasion |  |  |  |
| Yes | 4 (7.8) | 8 (7.5) | 1.000 |
| No | 47 (92.2) | 99 (92.5) |  |
| TNM stage |  |  |  |
| I,II | 22 (43.1) | 46 (43.0) | 1.000 |
| III,IV | 29 (56.9) | 61 (57.0) |  |
